# Comparative clinical and epidemiological characteristics of influenza and COVID-19 in hospitalized pediatric patients in Romania during and after the SARS-CoV-2 pandemic

**DOI:** 10.25122/jml-2026-0023

**Published:** 2026-04

**Authors:** Alina-Maria Rață, Anca Bălănescu, Valentina-Daniela Comănici, Tatiana Ciomârtan, Ioana-Florentina Codreanu, Gelu Onose

**Affiliations:** 1 Carol Davila University of Medicine and Pharmacy, Bucharest, Romania; 2 Alessandrescu Rusescu National Institute for Mother and Child Health, Bucharest, Romania; 3 Bagdasar Arseni Emergency Clinical Hospital, Bucharest, Romania

**Keywords:** COVID-19, influenza, acute respiratory failure, pneumonia, children

## Abstract

The COVID-19 pandemic significantly altered the epidemiology of respiratory viral infections in children. This study aimed to compare the clinical and epidemiological characteristics of influenza and COVID-19 infections in hospitalized pediatric patients in Romania during the pandemic. We conducted a retrospective observational study at the Alessandrescu-Rusescu National Institute for Mother and Child Health in Bucharest between January 1, 2020, and November 2025. Pediatric patients (0–18 years) hospitalized with COVID-19 infection or influenza A/B type were included. Demographic data, clinical presentation, comorbidities, complications, and outcomes were analyzed. A total of 234 patients with COVID-19 and 235 patients with influenza type A/B were included. The median age in the COVID-19 group was 13.9 months with a median length of hospitalization of 4 days, while in the influenza group, the median age was 32.6 months, with a median length of stay of 5 days. Influenza virus was associated with higher risks of intensive care unit admission, longer hospitalization (*P* < 0.001), pneumonia (22.0% vs 6.0%, *P* < 0.001), and acute respiratory failure (18.3% vs 10.7%, *P* = 0.025). In contrast to adult populations, influenza infection was associated with a more severe clinical course than COVID-19 in hospitalized pediatric patients, despite the younger age of COVID-19 patients. These findings emphasize the importance of preventive strategies against influenza in children.

## Introduction

The emergence of the novel coronavirus SARS-CoV-2 at the end of 2019 led to an unprecedented global health crisis, profoundly affecting healthcare systems and reshaping the epidemiology of infectious diseases worldwide [1]. To date, the World Health Organization (WHO) has reported 704 million confirmed cases of COVID-19 and 7,108,060 deaths worldwide [2]. Although children generally experienced milder COVID-19 than adults, the pandemic significantly influenced patterns of healthcare utilization, hospitalization criteria, and the circulation of other respiratory viruses [3].

Seasonal respiratory viruses, particularly respiratory syncytial virus (RSV) and influenza virus, have traditionally represented major causes of morbidity in the pediatric population [4]. Influenza infections are responsible each year for a substantial number of hospitalizations among young children, especially during the winter season, and may lead to complications such as pneumonia, respiratory failure, and subsequent bacterial infection [5-7]. The epidemiological behavior of influenza, characterized by well-defined seasonal peaks, had remained relatively stable prior to 2020 [8].

The implementation of public health measures during the COVID-19 pandemic- including social distancing, school closures, mask use, and quarantine strategies – led to a marked reduction in the transmission of most respiratory pathogens during the first year of the pandemic [9,10]. However, as restrictions were gradually lifted, atypical circulation patterns of respiratory viruses were, as expected, observed, including delayed or off-season peaks [11]. In parallel, the clinical spectrum of SARS-CoV-2 infection in children evolved over time, influenced by viral mutations, population immunity, and vaccination strategies [12].

To be more specific, for COVID-19, a series of public health measures were introduced: nationwide school closures from March to May 2020, mandatory mask-wearing in schools starting October 2020, intermittent social distancing measures during epidemic waves, and the initiation of the pediatric COVID-19 vaccination program in December 2021 for adolescents, with expansion to younger children in 2022 [13]. To reduce influenza transmission among children, seasonal influenza vaccination campaigns were conducted annually, typically in October-November, targeting children aged 6 months to 18 years.

While numerous studies have compared COVID-19 and influenza in adult populations, data directly evaluating these two infections in pediatric patients remain relatively limited. Understanding whether SARS-CoV-2 infections differ from influenza in terms of clinical severity, complications, and outcomes in children is essential for guiding preventative strategies, hospital resources, and vaccination policies.

Therefore, the aim of this study was to compare the epidemiological characteristics, clinical presentations, complications, and outcomes of these two respiratory infections in hospitalized children during the SARS-CoV-2 pandemic at a major pediatric center in Bucharest, Romania, and to evaluate potential differences in disease severity across distinct pandemic periods.

## Material and methods

We conducted a retrospective observational study at the Alessandrescu-Rusescu National Institute for Mother and Child Health in Bucharest. The study was conducted during the pandemic and post-pandemic period, from January 2020 to November 2025. We retrospectively enrolled pediatric patients (under 18 years old) who were admitted to our clinical institute for respiratory symptoms and were confirmed with COVID-19 or influenza type A/B infection. We excluded respiratory co-infections to obtain a clear view of the single infection. We also excluded patients with the age higher than 18 years old.

We collected demographic data, comorbidities, status of vaccination, and clinical features of the COVID-19 and, respectively, the influenza infections, such as: clinical manifestation (defined as upper respiratory tract infections (URTI), laryngitis, bronchiolitis, pneumonia or enterocolitis), complications (such as pneumonia, pleural effusion, empyema, abscess, need for intensive care admission, need for intubation and mechanical ventilation, sepsis or death) [14]. We recorded each patient’s hospitalization period.

The diagnosis was confirmed by real-time RT-PCR of respiratory samples (nasal swabs) or by a rapid antigen test for influenza A/B and COVID-19 [15].

We used descriptive statistics to summarize the epidemiological and related data, and the results were expressed as median (range). Categorical variables were coded numerically and compared using differential tests. For this purpose, normality was assessed using the Shapiro-Wilk test. Because several variables were not normally distributed (*P* < 0.05), nonparametric tests were used. Group comparisons were performed using the Mann–Whitney U test for continuous variables and Chi-square or Fisher’s exact test for categorical variables. Risk ratios (RRs) with 95% confidence intervals (CIs) were calculated for key complications. A *P* value of <0.05 was considered significant. For our statistical analysis, we used SPSS software v.20.

## Results

### Demographic characteristics

Between January 2020 and November 2025, 484 pediatric patients met the inclusion criteria. Of these, 234 patients were confirmed to have COVID-19, and 235 patients had influenza type A/B. Fifteen patients with confirmed coinfection were excluded from the analysis.

In the COVID-19 cohort, the median age of subjects was 13.9 months (range 1-180 months), suggesting that the study predominantly enrolled infants. The age distribution showed a pronounced asymmetry, with a mode of 1 month (the most frequent value), indicating that a large part of the sample was concentrated in the very young age group. In the influenza patient group, the median age of participants was 32.6 months (approximately 2.7 years; range 1–204 months), indicating that children hospitalized with COVID-19 were generally younger than those hospitalized with influenza. The difference was statistically significant (Mann–Whitney U test, *P* < 0.001).

Among influenza cases, 182 (77.4%) were diagnosed with influenza type A and 53 (22.6%) with influenza type B.

In the COVID-19 group, patients were male (61.5%), while in the influenza group, the gender distribution was practically equal (50.6%). Furthermore, in both studied groups, the majority of patients came from urban areas.

### Temporal and seasonal distribution

A distinct seasonal pattern was observed between the two types of infections: COVID-19 cases were most frequently recorded during summer (44.9%), while the influenza cases predominated in winter (77.9%).

A clear temporal shift in viral circulation was observed across pandemic phases. During 2020–2021, COVID-19 accounted for the vast majority of cases (93.2%), while influenza was nearly absent (6.8%). In 2022, following the relaxation of public health measures, influenza became predominant (75%), exceeding COVID-19 cases. During the post-restriction period (2023-2025), both viruses co-circulated, with a relatively balanced distribution between them (Table 1).

**Table 1:** Temporal distribution of COVID-19 and influenza cases across pandemic phases

Period	COVID-19 *n* (%)	Influenza *n* (%)	Total
2020–2021	55 (93.2%)	4 (6.8%)	59
2022	42 (25.0%)	126 (75.0%)	168
2023–2025	137 (56.6%)	105 (43.4%)	242

### Clinical characteristics

Underlying comorbidities were identified in 14 patients (5.9%) in the COVID-19 cohort and 29 (12.3%) in the influenza cohort. The prevalence of comorbidities differed significantly between the two groups (*P* = 0.024). The most frequent comorbidities for both groups included: asthma, cystic fibrosis, history of prematurity. Prematurity was defined as birth before 37 weeks of gestation. Most patients in this category were former preterm infants without bronchopulmonary dysplasia or other chronic lung diseases, representing otherwise healthy children with a history of prematurity.

Vaccination status was incompletely documented in the medical records. Among available data, none of the children were vaccinated against COVID-19, while influenza vaccination was documented in 5.1% (12) of cases.

**Figure 1:**
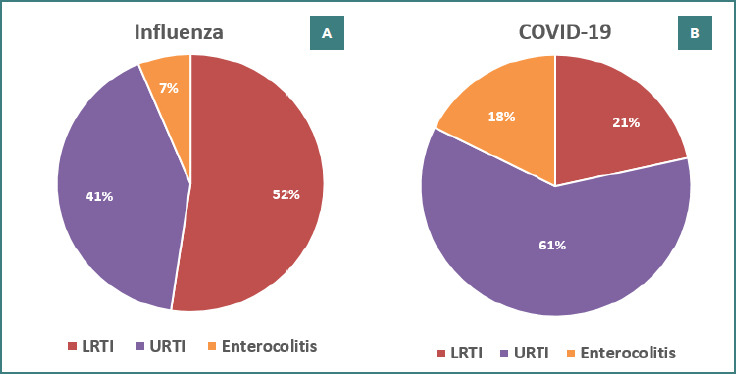
Clinical presentation among hospitalized children with influenza and COVID-19. A, Clinical presentation in children hospitalized with influenza. B, Clinical presentation in children hospitalized with COVID-19. **URTI, upper respiratory tract infection; LRTI, Lower respiratory tract infection**.

The clinical presentation differed notably between influenza and COVID-19 in hospitalized children. In the influenza group, lower respiratory tract infections (LRTI: including bronchiolitis, pneumonia, bronchopneumonia) were observed in 52.3% of cases, while upper respiratory tract infections (URTI: rhinitis, pharyngitis, laryngitis) were observed in 41.3%, and gastrointestinal symptoms in 6.4% (Figure 1A).

By contrast, patients with COVID-19 predominantly presented with URTI (60.3%), while LRTI occurred in 22.2% and enterocolitis in 17.5% (Figure 1B). The median length of stay in the hospital for the COVID-19 group was 4 days (range 0-30 days), while for the influenza cohort it was 5 days (range 0–34 days). This difference was statistically significant (Mann–Whitney U = 22304, *P* < 0.001), indicating a longer hospitalization period for patients with influenza than for the COVID-19.

The length of hospital stay (LOS) was analyzed across predefined pandemic phases in order to assess temporal changes in hospitalization patterns. For COVID-19 patients, during the 2020-2021 period, the median LOS was 5 days (IQR 3–7). In 2022, a decrease in LOS was observed, with a median of 4 days (IQR 3–5). This value remained stable during the 2023-2025 period, with a median LOS of 4 days (IQR 3–5).

**Figure 2:**
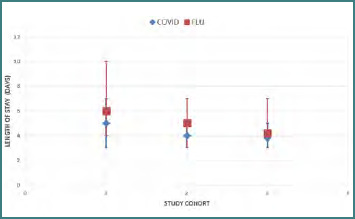
Comparison of the length of stay between the COVID-19 and influenza cohorts

For influenza cases, a similar but slightly more variable pattern was observed. During 2020-2021, influenza-related hospitalizations had a median LOS of 6 days, reflecting limited, but more severe cases during the period of restricted viral circulation. In 2022, when influenza became the predominant respiratory virus, the median LOS decreased to 5 days (IQR 3–7), while in 2023-2025 it further stabilized at 4 days (IQR 4–7; Figure 2).

Dot-and-whisker plot showing the median length of hospital stay (LOS) with min-max range for patients with COVID-19 and influenza across three study cohorts. Cohort 1 corresponds to 2020–2021, Cohort 2 to 2022, and Cohort 3 to 2023–2025.

Overall, a slight reduction in hospital stay duration was noted after the initial pandemic period, followed by stabilization in subsequent years without further significant variation.

### Complication and outcomes

Influenza infection was associated with a significantly higher risk of pneumonia than COVID-19 (RR = 3.77; 95% CI, 2.15–6.61; *P* < 0.001). After adjustment for underlying comorbidities using the Mantel–Haenszel method, influenza infection remained significantly associated with higher odds of pneumonia compared to COVID-19 (aOR = 4.17; 95% CI, 2.18–7.79). The association persisted after controlling for comorbidity status, thus suggesting an independent effect of influenza on pneumonia risk.

Moreover, a multivariate logistic regression was performed to assess the independent association of infection type with pneumonia, adjusting for age. COVID-19 was set as the reference category. Influenza infection was independently associated with a higher risk of pneumonia than COVID-19 (aOR = 3.7; 95% CI, 1.91–6.90; *P* < 0.001). After age adjustment, influenza infection remained independently associated with a higher risk of pneumonia, confirming that the observed difference between the two groups was not solely explained by the younger age of the COVID-19 cohort.

The risk of respiratory failure was significantly higher in the influenza group than in the COVID-19 cohort (RR = 1.71; 95% CI, 1.08–2.70; *P* = 0.025). After adjustment for underlying comorbidities using the same method as above, influenza infection remained significantly associated with a higher odds of respiratory failure than COVID-19 (aOR = 1.72; 95% CI, 1.03–2.87), indicating an independent effect of influenza on respiratory severity after controlling for comorbidities. Multivariable logistic regression was performed to assess the association between infection type and pneumonia, adjusting for age. COVID-19 was set as the reference category. The results showed that influenza infection was independently associated with a higher risk of respiratory failure compared with COVID-19 (aOR = 2.02; 95% CI, 1.17–3.49; *P* = 0.012). Age was not a significant predictor in this model (aOR = 0.996; 95% CI, 0.987–1.004; *P* = 0.300).

A higher proportion of cases were complicated by sepsis in the influenza group (3%) than in the COVID-19 group (0.9%), although this difference did not reach statistical significance (*P* = 0.17; Table 2).

**Table 2:** Comparison of the length of stay between the COVID-19 and influenza cohorts

Complication	COVID-19 *n* (%)	Influenza *n* (%)	*P*
Pneumonia	14 (6)	53 (22.6)	<0.001
Otitis	4 (1.7)	34 (14.5)	<0.001
Respiratory failure	25 (10.7)	43 (18.3)	0.025
Pleural effusion	2 (0.9)	7 (3.0)	0.17
Drained pleural effusion	2 (0.9)	3 (1.3)	1.00
Pneumothorax	1 (0.4)	1 (0.4)	1.00
Abscess	1 (0.4)	1 (0.4)	1.00
Meningitis	0 (0)	1 (0.4)	1.00
Sepsis	2 (0.9)	7 (3.0)	0.17
Intubation and mechanical ventilation	3 (1.3)	5 (2.1)	0.73
Death	0 (0)	2 (0.9)	0.157

Intensive care admission with the need for intubation and mechanical ventilation was required in 2.1% (*n* = 5) of patients with influenza and 1.3% (*n* = 3) with COVID-19. Among influenza patients receiving ventilator support, 40% (*n* = 2) died, whereas none died among ventilated COVID-19 patients. These findings highlight that severe respiratory involvement requiring mechanical ventilation was more frequent and associated with higher mortality in influenza compared to COVID-19.

Regarding mortality, there were two deaths in the influenza group, while none occurred among patients with COVID-19; however, the difference did not reach statistical significance (*P* = 0.157).

### Comparison between pandemic periods (COVID-19 subgroup analysis)

Given that the influenza infection had more complications, at least for our pediatric patients, we also considered the different phases of the COVID-19 pandemic. Throughout the pandemic, the virus has undergone multiple mutations, leading to the emergence of new strains with distinct clinical characteristics. Therefore, we assessed the severity of COVID-19 infections in children across two time periods: the beginning of the pandemic in 2020–2021, when early strains predominated, and the period between 2022–2025 (mainly after the end of the pandemic).

When comparing two COVID-19 infection-related periods (the pandemic 2020–2021 vs. post-pandemic 2022–2025), respiratory failure was significantly more frequent during the early phase of the pandemic: in 2020–2021, 18.5% of patients developed respiratory failure compared to only 8.3% during 2022–2025 (*P* < 0.05).

Furthermore, intubation and mechanical ventilation were required in 5.6% of patients during 2020–2021, whereas no cases required intubation during 2022–2025, suggesting a decrease in disease severity in later pandemic and post-pandemic stages (*P* = 0.01).

## Discussion

Our comparative approach, as detailed above, enabled us to identify the clinical features of both infections: COVID-19 and influenza A/B. It is important to emphasize that, to our knowledge, there are limited bibliographic resources to directly compare these two infections in children, whereas numerous such articles exist in the literature regarding adults. Moreover, we identified key differences in the features of COVID-19 and influenza in children compared with adults.

One finding in our study was that the majority of patients in the COVID-19 and influenza groups were male gender. This is consistent with previous studies, which found that COVID-19 and influenza have a higher incidence among male patients than among female patients [16,17]. Although studies in the adult population suggested that the male gender is a risk factor for more frequent need for hospitalization and complications [18,19], our study did not confirm this finding.

Another important observation from the comparison of the two infections was a statistically significant difference in age distribution between the groups. The median age of COVID-19 patients in our study was approximately 1 year, suggesting that most were infants. Meanwhile, the median age in the influenza group was 2.7 years. This finding may reflect differences in viral epidemiology and host susceptibility during the pandemic. Younger children, particularly infants, were more frequently hospitalized with COVID-19, possibly due to precautionary admission criteria and limited prior immunity in this age group during the early pandemic waves. In contrast, influenza has traditionally affected a broader age range in children, with higher hospitalization rates among toddlers and preschool-age children. Importantly, age is a potential confounding factor in severity analyses, as younger children are generally at higher risk for respiratory complications. Therefore, the significant age difference between groups was taken into account when interpreting comparative outcomes, such as respiratory failure and pneumonia.

Compared to other clinical studies, the median age for COVID-19 and the influenza groups appears to be much lower in our study; specifically, the median age for COVID-19 infection in the literature is 5 years [20] and 8 years for influenza [21]. These findings may be due to the fact that pediatric patients of younger age have a tendency to have a more complicated course of the disease and therefore are admitted more easily to the hospital, whereas older children manage to be treated at home, and since our study was conducted on hospitalized patients only, we just reviewed patients with a lower age.

It has been important to assess comorbidities in both groups to identify patients at risk for more severe disease. In our study, the COVID-19 group had fewer comorbidities than the influenza group. This may be explained by the fact that children with chronic pulmonary conditions, including cystic fibrosis, generally exhibit higher preventive measures, such as mask use and social distancing, implemented during the initial waves of COVID-19, compared with the general pediatric population [22]. These behavioral measures may have contributed to the relatively mild outcomes observed in this subgroup. In the literature, a large cohort study found that COVID-19 patients had fewer comorbidities than the influenza group in the USA, but more comorbidities in South Korea [23], suggesting that results may differ by country, region, or clinical setting [24]. Moreover, we observed that the main comorbidities were chronic respiratory diseases, such as asthma or a history of prematurity. This finding is consistent with previous reports regarding these two infections in children and in adults [25,26].

Regarding vaccination, none of the children in our study received a COVID-19 vaccine, and only 12 (5.1%) received the influenza vaccine. In Romania, the influenza vaccine is not included in the National Immunization Program, but it is recommended and fully reimbursed for the pediatric population. A major policy change was implemented in 2023 (Ministerial Order no. 3120/2023), extending full reimbursement of the influenza vaccine to all children through general practitioner prescription, whereas prior to this, reimbursement was primarily targeted to high-risk groups. This regulatory change may introduce bias in the interpretation of our findings over the given timeline, as it could have influenced the proportion of vaccinated children and, consequently, the clinical presentation and severity of influenza cases.

In Romania, COVID-19 vaccination has never been included in the National Immunization Program but has been recommended through successive governmental decisions. Vaccination for children was introduced progressively: in 2021 for those aged 12 and older, on March 31, 2022, for children aged 5–11, and in 2023 for younger children, including infants older than 6 months. Since 2024, there has been a government decision (HG 1640/2024) that institutionalizes the vaccination protocol in Romania, stating that vaccines can be administered by a general practitioner and recorded in RENV (National Electronic Database of Immunization). However, national surveys suggest relatively low parental acceptance of pediatric COVID-19 vaccination, with approximately 11% of parents reporting that their 12–18-year-old children have been vaccinated in one regional study [27].

The duration of hospitalization was similar in both groups in our study, with median lengths of stay of 4 days for COVID-19 and 5 days for influenza. In a systematic review of the clinical features of COVID-19 in children, the median length of stay was 9 days [28,29], whereas, in the literature, the median hospital length of stay for influenza was 3 days [30], which differs from our finding. The difference in the length of stay for children with COVID-19 between our study and the literature may stem from the fact that the majority of children with COVID-19 were infants with mild symptoms, especially in 2022–2025. The systematic review evaluating the length of stay for children with COVID-19 was conducted during the first years of the pandemic (2020–2022), when the initial SARS-CoV-2 strain was associated with a more severe course of the disease [31,32], thereby contributing to a longer hospitalization.

The analysis of hospital length of stay across pandemic phases demonstrated a progressive reduction in hospitalization days for both COVID-19 and influenza infections. For COVID-19, LOS was higher during the initial pandemic phase (median 5 days) and decreased in 2022, followed by stabilization at 4 days in the post-restriction period (2023–2025). A similar trend was seen for influenza, with LOS decreasing from 6 days in 2020–2021 to 5 days in 2022 and 4 days in 2023–2025. This downward trend likely reflects a combination of factors, including improved clinical experience, earlier presentation and diagnosis, and evolving hospitalization criteria after the acute phase of the pandemic. In addition, increasing population immunity and changes in the epidemiology of SARS-CoV-2 may have contributed to milder clinical presentations.

Another finding in our study is the epidemiology of the two infections, considering the distribution of cases throughout the year. While the influenza virus continued to show peaks of infection during winter and spring, the SARS-CoV-2 virus had its peak during the summer, followed by winter, a finding consistent with other studies [33,34]. Also, the lower number of cases during 2020–2021 reflects reduced hospital admissions and decreased viral transmission during that period, due to strict public health measures. Moreover, our findings demonstrate that classical seasonality patterns were disrupted during the pandemic, with atypical resurgence of influenza during 2022, consistent with observations in previous studies [35].

Assessing the severity of the disease, the overall course of the infections was more severe in the influenza group with a longer period of hospitalization, more frequent admissions in the intensive care unit, need for oxygen therapy and invasive ventilation, and, respectively, complications towards pneumonia and sepsis when compared to the COVID-19 infection. After adjusting for age and comorbidities, the results show that differences in outcomes between the two infections are not solely due to differences in age distribution or comorbidities between the cohorts.

In contrast to our results, previous studies showed that patients infected with SARS-CoV-2 were at a higher risk of developing disease complications than those infected with influenza [23,36]. It is important to emphasize that the studies mentioned earlier were conducted on the adult population. Thus, although it is generally considered that COVID-19 has a severe course in the adult population, we observed and demonstrated that, in children, it typically presents as an upper respiratory tract infection (URTI) with minimal complications. Therefore, the influenza virus remains one of the main viruses causing severe disease in children, thereby leaving children, a vulnerable group, in need of continued protection.

Regarding the clinical manifestations of the two respiratory infections, our study revealed that, in both conditions, the main clinical presentation was upper respiratory tract infection (URTI), characterized by fever, cough, and fatigue. This is consistent with the literature [17,37]. It is important to note that the most frequent symptoms in the influenza group were followed by pneumonia, whereas in the COVID-19 group, the subsequent symptoms were gastrointestinal, followed by bronchiolitis, the main manifestation in the infant population, as noted in the literature [38,39]. A retrospective case-control study conducted in the first years of the pandemic showed that dyspnea and gastrointestinal symptoms were more frequent in COVID-19 than in the flu [36], supporting the related finding from our study. A special remark regarding influenza: the second most common manifestation was pneumonia, highlighting the risk of subsequent bacterial infection in this group.

Our analysis of pandemic periods within the COVID-19 cohort revealed that respiratory complications were more frequent during the early stages of the pandemic, whereas later periods were characterized by milder clinical presentations. This trend likely reflects immunity and improvements in clinical management protocols over time [40].

Our study is subject to several limitations. Firstly, the screening algorithm at our center differed between seasonal influenza and SARS-CoV-2. During the pandemic (2020–2022), according to the protocol, all patients admitted to the hospital were tested for COVID-19, while only patients with typical flu symptoms were tested for influenza. This may result in a larger number of COVID-19 patients with milder symptoms or subclinical infections. Secondly, younger age and fewer comorbidities in COVID-19 patients admitted to our hospital compared to those with influenza may also reflect a lower threshold for admitting patients with SARS-CoV-2 infection, due to challenges with quarantine and limited data on reliable predictors of disease severity, especially in the early phases of the pandemic. Likewise, mild cases that did not require hospitalization may have been underreported, especially for influenza. Additionally, although our analyses adjusted for comorbidities, other factors, such as socio-economic status, prior immunization status, or access to healthcare, may have influenced the results. Nevertheless, the large sample size and the comparative approach strengthen the validity of our findings.

Despite limitations, our study provides relevant insights into the common clinical features and differences between pediatric patients with COVID-19 and those with seasonal influenza.

## Conclusion

In this retrospective study of hospitalized pediatric patients, influenza infection was associated with a more severe clinical course compared to COVID-19 during both the pandemic and post-pandemic periods. Children with influenza showed a significantly higher risk of pneumonia and respiratory failure, along with an increased need for intensive care support and consequent longer hospitalization. In contrast, COVID-19 was predominantly characterized by milder respiratory forms, particularly in the later phases of the pandemic.

These findings highlight the ongoing clinical relevance of seasonal influenza in the pediatric population and reinforce the importance of preventive strategies, including vaccination, to reduce severe outcomes, particularly in the post-pandemic era of co-circulating respiratory viruses.

## References

[1] Zhang C, Gu J, Chen Q, Deng N, Li J, Huang L, Zhou X (2020). Clinical and epidemiological characteristics of pediatric SARS-CoV-2 infections in China: A multicenter case series. PLoS Med.

[2] Worldometer COVID-19 coronavirus pandemic. [Internet].

[3] Dao TL, Hoang VT, Colson P, Million M, Gautret P (2021). Co-infection of SARS-CoV-2 and influenza viruses: A systematic review and meta-analysis. J Clin Virol Plus.

[4] Aghbash PS, Eslami N, Shirvaliloo M, Baghi HB (2021). Viral coinfections in COVID-19. J Med Virol.

[5] Steponavičienė A, Burokienė S, Ivaškevičienė I, Stacevičienė I, Vaičiūnienė D, Jankauskienė A (2023). Influenza and Respiratory Syncytial Virus Infections in Pediatric Patients during the COVID-19 Pandemic: A Single-Center Experience. Children (Basel).

[6] Uyeki TM, Hui DS, Zambon M, Wentworth DE, Monto AS (2022). Influenza. Lancet.

[7] Stowe J, Tessier E, Zhao H, Guy R, Muller-Pebody B, Zambon M, Andrews N, Ramsay M, Lopez Bernal J (2021). Interactions between SARS-CoV-2 and influenza, and the impact of coinfection on disease severity: a test-negative design. Int J Epidemiol.

[8] Chotpitayasunondh T, Fischer TK, Heraud JM, Hurt AC, Monto AS, Osterhaus A (2021). Influenza and COVID-19: What does co-existence mean?. Influenza Other Respir Viruses.

[9] She J, Liu L, Liu W (2020). COVID-19 epidemic: Disease characteristics in children. J Med Virol.

[10] Liu P, Xu M, Cao L, Su L, Lu L, Dong N (2021). Impact of COVID-19 pandemic on the prevalence of respiratory viruses in children with lower respiratory tract infections in China. Virol J.

[11] Kondo Y, Miyazaki S, Yamashita R, Ikeda T (2020). Coinfection with SARS-CoV-2 and influenza A virus. BMJ Case Rep.

[12] Swets MC, Russell CD, Harrison EM, Docherty AB, Lone N, Girvan M, Hardwick HE; ISARIC4C Investigators; Visser LG, Openshaw PJM, Groeneveld GH, Semple MG, Baillie JK (2022). SARS-CoV-2 co-infection with influenza viruses, respiratory syncytial virus, or adenoviruses. Lancet.

[13] Horga NG, Cirnatu D, Kundnani NR, Ciurariu E, Parvu S, Ignea AL, Borza C, Sharma A, Morariu S (2022). Evaluation of non-pharmacological measures implemented in the management of the COVID-19 pandemic in Romania. Healthcare (Basel).

[14] Ma S, Lai X, Chen Z, Tu S, Qin K (2020). Clinical characteristics of critically ill patients co-infected with SARS-CoV-2 and the influenza virus in Wuhan, China. Int J Infect Dis.

[15] Cox NJ, Subbarao K (2000). Global epidemiology of influenza: past and present. Annu Rev Med.

[16] Wang XL, Yang L, Chan KH, Chan KP, Cao PH, Lau EH (2015). Age and Sex Differences in Rates of Influenza-Associated Hospitalizations in Hong Kong. Am J Epidemiol.

[17] Karagiannidis C, Mostert C, Hentschker C, Voshaar T, Malzahn J, Schillinger G (2020). Case characteristics, resource use, and outcomes of 10 021 patients with COVID-19 admitted to 920 German hospitals: an observational study. Lancet Respir Med.

[18] Scully EP, Haverfield J, Ursin RL, Tannenbaum C, Klein SL (2020). Considering how biological sex impacts immune responses and COVID-19 outcomes. Nat Rev Immunol.

[19] Khorramdelazad H, Kazemi MH, Najafi A, Keykhaee M, Zolfaghari Emameh R, Falak R (2021). Immunopathological similarities between COVID-19 and influenza: Investigating the consequences of Co-infection. Microb Pathog.

[20] Christophers B, Gallo Marin B, Oliva R, Powell WT, Savage TJ, Michelow IC (2022). Trends in clinical presentation of children with COVID-19: a systematic review of individual participant data. Pediatr Res.

[21] Osterholm MT, Kelley NS, Sommer A, Belongia EA (2012). Efficacy and effectiveness of influenza vaccines: a systematic review and meta-analysis. Lancet Infect Dis.

[22] Flume PA, Saiman L, Marshall B (2022). The Impact of COVID-19 in Cystic Fibrosis. Arch Bronconeumol.

[23] Burn E, You SC, Sena A, Kostka K, Abedtash H, Abrahao MTF (2020). Deep phenotyping of 34,128 patients hospitalised with COVID-19 and a comparison with 81,596 influenza patients in America, Europe and Asia: an international network study. medRxiv [Preprint].

[24] Cobb NL, Sathe NA, Duan KI, Seitz KP, Thau MR, Sung CC (2021). Comparison of Clinical Features and Outcomes in Critically Ill Patients Hospitalized with COVID-19 versus Influenza. Ann Am Thorac Soc.

[25] Sanchez-Ramirez DC, Mackey D (2020). Underlying respiratory diseases, specifically COPD, and smoking are associated with severe COVID-19 outcomes: A systematic review and meta-analysis. Respir Med.

[26] Bai Y, Tao X (2021). Comparison of COVID-19 and influenza characteristics. J Zhejiang Univ Sci B.

[27] Manolescu LSC, Zaharia CN, Dumitrescu AI, Prasacu I, Radu MC, Boeru AC (2022). COVID-19 Parental Vaccine Hesitancy in Romania: Nationwide Cross-Sectional Study. Vaccines (Basel).

[28] Yasuhara J, Kuno T, Takagi H, Sumitomo N (2020). Clinical characteristics of COVID-19 in children: A systematic review. Pediatr Pulmonol.

[29] Teich VD, Klajner S, Almeida FAS, Dantas ACB, Laselva CR, Torritesi MG (2020). Epidemiologic and clinical features of patients with COVID-19 in Brazil. Einstein (Sao Paulo).

[30] Kwong KL, Lung D, Wong SN, Que TL, Kwong NS (2009). Influenza-related hospitalisations in children. J Paediatr Child Health.

[31] Robu AM, Onose G, Ulinici MT, Rață A, Bălănescu A, Comănici VD, Ciomârtan T, Codreanu IF (2024). Actual data regarding the impact of viral respiratory co-infection (COVID-19 and flu/Respiratory Syncytial Virus-RSV): a systematic review. Balneo and PRM Research Journal.

[32] Brehm TT, van der Meirschen M, Hennigs A, Roedl K, Jarczak D, Wichmann D (2021). Comparison of clinical characteristics and disease outcome of COVID-19 and seasonal influenza. Sci Rep.

[33] Ladhani SN, Amin-Chowdhury Z, Davies HG, Aiano F, Hayden I, Lacy J (2020). COVID-19 in children: analysis of the first pandemic peak in England. Arch Dis Child.

[34] Rus MA, Leucuța DC, Briciu VT, Muntean MI, Filip VP, Ungureanu RF (2025). Influenza A vs. COVID-19 A Retrospective Comparison of Hospitalized Patients in a Post-Pandemic Setting. Microorganisms.

[35] Miron VD, Bar G, Filimon C, Craiu M (2022). From COVID-19 to Influenza-Real-Life Clinical Practice in a Pediatric Hospital. Diagnostics (Basel).

[36] Tang X, Du RH, Wang R, Cao TZ, Guan LL, Yang CQ (2020). Comparison of Hospitalized Patients With ARDS Caused by COVID-19 and H1N1. Chest.

[37] Varshney K, Pillay P, Mustafa AD, Shen D, Adalbert JR, Mahmood MQ (2023). A systematic review of the clinical characteristics of influenza-COVID-19 co-infection. Clin Exp Med.

[38] Wang D, Hu B, Hu C, Zhu F, Liu X, Zhang J (2020). Clinical Characteristics of 138 Hospitalized Patients With 2019 Novel Coronavirus-Infected Pneumonia in Wuhan, China. JAMA.

[39] Cao B, Li XW, Mao Y, Wang J, Lu HZ, Chen YS (2009). ; National Influenza A Pandemic (H1N1) 2009 Clinical Investigation Group of China. Clinical features of the initial cases of 2009 pandemic influenza A (H1N1) virus infection in China. N Engl J Med.

[40] Tsabouri S, Makis A, Kosmeri C, Siomou E (2021). Risk Factors for Severity in Children with Coronavirus Disease 2019: A Comprehensive Literature Review. Pediatr Clin North Am.

